# Research in the Field of Drug Design and Development

**DOI:** 10.3390/ph16091283

**Published:** 2023-09-11

**Authors:** Grazyna Biala, Ewa Kedzierska, Marta Kruk-Slomka, Jolanta Orzelska-Gorka, Sara Hmaidan, Aleksandra Skrok, Jakub Kaminski, Eva Havrankova, Dominika Nadaska, Ivan Malik

**Affiliations:** 1 Chair and Department of Pharmacology with Pharmacodynamics, Medical University of Lublin, Chodźki 4A, 20-093 Lublin, Poland; ewa.kedzierska@umlub.pl (E.K.); marta.kruk@umlub.pl (M.K.-S.); jolanta.orzelska-gorka@umlub.pl (J.O.-G.);; 2 Department of Chemical Drugs, Faculty of Pharmacy, Masaryk University of Brno, 601 77 Brno, Czech Republic; havrankovae@pharm.muni.cz; 3 Department of Pharmaceutical Chemistry, Faculty of Pharmacy, Comenius University Bratislava, 832 32 Bratislava, Slovakiamalik@fpharm.uniba.sk (I.M.)

**Keywords:** drug discovery, drug synthesis, in vivo studies, in vitro studies, clinical trials

## Abstract

The processes used by academic and industrial scientists to discover new drugs have recently experienced a true renaissance, with many new and exciting techniques being developed over the past 5–10 years alone. Drug design and discovery, and the search for new safe and well-tolerated compounds, as well as the ineffectiveness of existing therapies, and society’s insufficient knowledge concerning the prophylactics and pharmacotherapy of the most common diseases today, comprise a serious challenge. This can influence not only the quality of human life, but also the health of whole societies, which became evident during the COVID-19 pandemic. In general, the process of drug development consists of three main stages: drug discovery, preclinical development using cell-based and animal models/tests, clinical trials on humans and, finally, forward moving toward the step of obtaining regulatory approval, in order to market the potential drug. In this review, we will attempt to outline the first three most important consecutive phases in drug design and development, based on the experience of three cooperating and complementary academic centers of the Visegrád group; i.e., Medical University of Lublin, Poland, Masaryk University of Brno, Czech Republic, and Comenius University Bratislava, Slovak Republic.

## 1. Introduction

Through the process of drug design and discovery, potential new therapeutic agents are identified, using different computational, experimental, and clinical models [1,2,3]. Despite advances in biotechnology and pharmacology, and in our understanding of biological mechanisms, drug discovery is still a lengthy, costly, difficult, and sometimes inefficient process. In its first step, drug design involves the outline of compounds that are complementary in shape and structure to the molecular target with which they interact and bind. Nowadays, drug design frequently relies on computer modeling techniques and bioinformatic approaches. That means that a synthetic approach has to already exist, or has to be developed according to the natural physico-chemical properties of the reacting compounds. The environmental burden, production safety, economic point of view, and other principles of green chemistry (see Section 2) should be taken into account when designing methods for the synthesis of target compounds.

In general, the process of drug development consists of three main stages: drug discovery, pre-clinical development using cell-based and animal models/tests, clinical trials on humans and, finally, moving forward toward the step of obtaining regulatory approval, in order to market the potential drug (see, e.g., ref. [4] for drug screening). Modern drug discovery aims to increase the affinity, selectivity (to reduce the potential of side effects), efficacy/potency, metabolic stability (to increase the half-life), and oral bioavailability of new drugs. As stated, drug discovery starts with the finding of a hit molecule that elicits a desired activity in a screening assay [1,2,4]. Then, its structure is optimized, in terms of improving its affinity and selectivity, reducing its toxicity, improving its water and lipid solubility, and improving its pharmacokinetic properties in general, which converts the hit molecule into a lead one; i.e., a drug candidate. Next, pre-clinical studies are focused on establishing the mode of action of the drug candidate, and its pharmacokinetics in animals (rodents, zebrafish), including its bioavailability, toxic metabolites, if any, routes of excretion, efficacy on animals, drug formulation, and stability. Thirdly, the clinical trials comprise the longest and the most expensive stage of the process, consisting of three phases; i.e., on healthy volunteers, and then on several hundred patients suffering from the target disease, and then several thousand patients from several clinical centers around the world are involved. The aim of this phase is to evaluate the efficacy and safety of the drug in humans, its pharmacokinetics in the human body, and the immediate side effects, if there are any [3]. If the drug passes this phase successfully, then it is ready for registration and marketing. However, the drug continues to be observed for its safety and side effects. This last phase is known as post-marketing surveillance, and it is practically endless, continuing until the drug is on the market.

In this review, we will attempt to outline the first three most important consecutive phases in drug design and development, based on the experience of three cooperating and complementary academic centers of the Visegrád group. The elements that make up the legal and legislative regulations aimed at registering a drug and introducing it onto the market may be the subject of other review works in this field. In our paper, we focused especially on small-molecule drugs, due to the significant amount of information collected on this extremely important topic, and the experiences of the authors, and the partners of the international project in this field of research. Several innovative types of therapeutics were also briefly discussed. Our intention was to use our own scientific and academic experiences from three complementary centers and cooperating scientists and, in addition, briefly inform about the design and development of several classes of non-small-molecule drugs, as well. Several types of these innovative therapeutics were discussed as the topics of open lectures organized within the project for students, scientists, and the general public.

## 2. Principles of Green Chemistry—A New Approach to the Synthesis of Drugs

**Figure 1:**
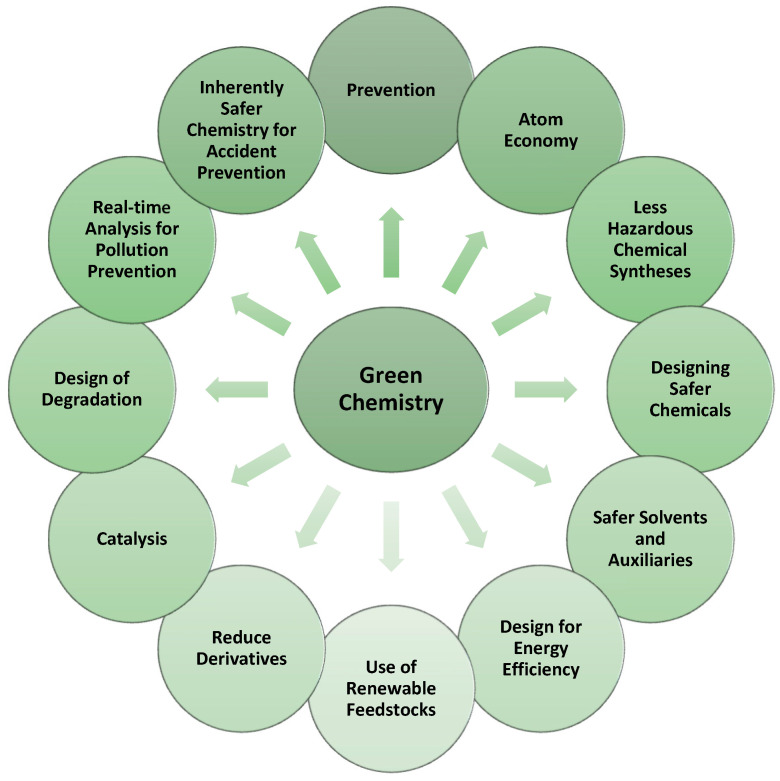
The Twelve Principles of Green Chemistry, inspired by [6].

Chemistry is all around us. It is used in the food industry, materials, electronics, cosmetics, and many other industries. Such a wide use of chemistry, however, can only be sustainable if we try to minimize its negative impact on the environment. With this aim, in the 1990s, Dr. John Warner and Dr. Paul Anastas developed the Twelve Principles of Green Chemistry [5]. Green chemistry, by definition, is the design of chemical products and processes that reduce and/or eliminate the use or generation of hazardous substances [5,6]. Nowadays, these principles are adapted in the vast majority of chemical laboratories, and they are taken into account when designing new compounds, to minimize the risk from chemical reactions to human health and the environment. The design and production of new pharmaceuticals are no exception (Figure 1).

One of the most interesting principles of green chemistry, which was very quickly and effectively applied to the synthesis of organic compounds, is the catalysis of these reactions. The reactions of organic compounds often do not proceed with a 100% conversion of the starting substances, large amounts of undesirable by-products can be formed, reactions require extreme conditions, etc. All these problems are incompatible with green chemistry (atom economy, reducing derivatives, energy efficiency, etc.). However, they can have a relatively simple solution—catalysis [6,7,8,9].

A catalyst is defined as “a substance that changes the velocity of a reaction without itself being changed in the process” [5]. This means that the catalyst reduces the activation energy of the reaction, i.e., the minimum energy that the particles of the reacting substances must-have in order for a chemical reaction to occur (for example, to create one new substance from two reacting compounds). At the same time, the catalyst emerges from the entire reaction in its original, unchanged form. Theoretically, this means that the catalyst can be used in small quantities, and be recycled indefinitely; therefore, it does not generate any waste [7,8,9]. Of course, theory is not practice. Therefore, catalysts and their use have been studied very intensively in recent decades, to improve the selectivity and yield of the reactions in which they are used, as well as the recyclability, economy, and safety of catalysts.

One of the most common and, at the same time, most widely used ways to obtain highly recyclable, economical, efficient, and selective catalysts is the immobilization of catalysts on heterogeneous supports, such as polymers [10,11], oxides [12,13,14], and others [15,16,17,18,19]. For example, enzymes are nowadays widely used in the manufacturing of pharmaceuticals, fine chemicals, flavors, food, fragrances, and other products [19]. Therefore, scientists have also focused on how best to immobilize them to increase their efficiency and other desirable properties. Recently, some interesting new ways of immobilizing them were published.

Enzymes immobilized on DNA nanostructures can potentially be used in the synthesis of complex biomolecules, which cannot be realized through the conventional synthetic approach [20]. Very interesting groups of supports comprise agriculture and food waste [19,21]. These materials usually have a high surface area and various functional groups (hydroxyl-, carboxyl-, amino-, thiol-, and others) available for immobilization [19,21]. Examples of successfully used supporters from this group are eggshell [21,22], eggshell membrane [21,23,24,25], coconut fiber [21,26,27,28], corn cob [21,29], corn husk [21,30], rice husk [21,27,31,32], spent coffee grounds [21,33,34], spent grains [21,35], and so on.

Another remarkable class of supports for the immobilization of enzymes are MOFs (metal–organic frameworks). These compounds consist of metal ions (or clusters) coordinated with organic ligands, to form a crystalline structure similar to a cage [36]. The structure of a MOF can be relatively easily modified to reach a very high surface area and a high porosity—this leads to a very high loading of enzymes and, therefore, a very good biocatalytic activity [15,21]. Furthermore, with the right choice of particle size and other related properties, we can also obtain an easily recyclable catalytic system—MOFs can be separated from the reaction mixture via, for example, filtration or centrifugation [37].

Enzymes supported on magnetic nanoparticles can also be easily separated from the reaction mixture using an external magnetic field. Iron oxide (Fe_3_O_4_) is most widely used as the magnetic supporter for the following reasons: its nontoxicity, high biocompatibility, and easy-to-handle immobilization [38,39,40,41]. However, other magnetic particles, such as silica-coated magnetic nanocarriers [41], magnetic amine-functionalized nanospheres [42], and chitosan-modified Fe_3_O_4_ nanoparticles [43], can also be used.

## 3. Brief Insight into Development and Optimization in Drug Discovery, and Fundamental Roles and Importance of Computer-Aided Drug Design in This Process

Early-phase drug discovery is focused on finding highly promising synthetic compounds, semi-synthetic derivatives, or molecules of natural origin that show a notable impact on various phases of a disease, by controlling particular biological signaling cascade(s) in desirable ways. From the point of view of medicinal chemistry, this process is pretty complex, is quite often very time-consuming, and involves many theoretical, as well as experimental investigations, and many optimization steps within particular fields of this scientific discipline [44,45].

The platforms for optimization include the identification and proper characterization of relevant biological targets: systematic screening, the very precise evaluation of ligand (mono- or multi-functional drug)–target interactions, and rational drug design, as well as the detailed investigation and correct interpretation of structure–activity, structure–pharmacokinetics, and/or structure–toxicity relationships [44,46].

The integral part of this optimization is defining the pharmacophore as a very essential feature of the drug responsible for the desired biological activity, as well as suitably chosen bioisosteric modifications with the implementation of the principles and rules of bioisosterism.

Bioisosterism means the replacement of particular groups within the structure of a biologically active compound, in order to improve its biological activity, selectivity toward specific biological target(s), or metabolic stability, or to achieve a decrease in toxicity, if selective toxic properties are not therapeutically required [47].

Other optimization procedures aim to suitably modify the selectivity profiles of drugs toward chosen biological targets, with regard to the desired biological activity, and property-based design, including the structural, physicochemical, and pharmacokinetic (what the body does to the drug) features of the drugs, or promising drug candidates [44,45]. Furthermore, their biotransformation pathways in vivo, and elimination routes from the organism, as well as strategies for drug repurposing, as identified new therapeutic areas for previously approved or investigational drugs that are outside the scope of the original medical indication [44,48], definitely have to be taken into consideration.

The design and structural optimization of drugs using so-called proteolysis-targeting chimera (PROTAC) technology [46] marks a remarkably innovative conceptual shift from the views reflecting traditional concepts in drug discovery, based on the inhibition of biological functions by small-molecule drugs.

However, several more- or less-serious obstacles, or even failures, can occur at each level of the drug discovery and development process. Moreover, the financial requirements and excessively long time connected with bringing a biologically effective, selective, and non-toxic compound drug to the pharmaceutical and medicinal market are regarded as other possible areas where the situation can become complicated. Thus, computer-aided drug design (CADD) has been viewed as a very powerful strategy in the drug discovery pipeline. Structure-based and ligand-based drug design techniques via CADD provide essential information for molecular docking (MD), molecular dynamics (MDy), and ADMET (absorption, distribution, metabolism, elimination, and toxicity) modeling, respectively [44,49,50].

The first two areas (MD and MDy) may provide a very beneficial look into the efficacy or potency of drugs; the latter is capable of notably influencing the clinical success of the drugs involved in clinical trials. The aim of medicinal chemists is to precisely design and synthesize safe drugs showing a favorable combination of pharmacodynamic (what the drug does to the body), structural, physicochemical, pharmacokinetic (ADME features), and toxicological variables [51,52]. MD is a computational technique aimed at finding the accurate binding pose of a biological target (protein)–ligand (drug) complex, and evaluating and describing the strength of such a complex, by using various scoring functions and parameters to select the best pose generated by each molecule in a rank order [49].

MDy simulation can shed light on the prediction of atom movement in a molecular/biological system. This timely process is based on intermolecular interactions, following Newtonian physics [52]. Both the recognition and the capture of the motion and position of each atom in the system provide extremely beneficial information to scientists. For example, the simulations could contribute to uncovering the mystery around progressive neurodegenerative diseases caused by various types of protein misfolding and aggregation. Alzheimer’s disease is characterized by the formation of amyloid plaques both extracellularly (β-amyloid peptide) and intracellularly (tau protein); the hallmark of Parkinson’s disease is an accumulation of aggregates of the α-synuclein protein [49,53,54].

Small molecules are (organic) synthetic compounds or molecules of natural origin, which have a molecular weight (*MW*) < 1500 Da (or in g/mol units). The value could not be viewed so strictly, several scientific papers regarded the interval 900–1000 Da as the upper (fixed) limit [55,56], in fact. The *MW* descriptor correlates with the ability of the compounds:

(a) rapidly cross biological membranes;
(b) gain access to the relevant intracellular biological targets (the DNA or RNA of proteins, for example).

The impact of drugs on these biological targets is often selective, dose-dependent, and associated with strongly required pharmacotherapeutic interventions (the treatment of particular diseases) or unfavorable effects (carcinogenic, teratogenic, etc.).

Not only are CADD approaches frequently utilized in evaluating structurally relatively simple small-molecule drugs and drug candidates, but these computational techniques are also employed in the investigation of peptide and protein therapeutics [57,58], PROTACs [46,59], and nucleic-acid-based therapeutics, as small interfering RNAs (siRNAs), for example, are [60,61,62].

Peptides contribute notably to advances in the fields of pharmacy and medicine. The successful design of peptide and protein therapeutics is dependent on knowledge about their structure and their biological targets. The proper identification of active sites for the proteins is considered a preliminary task to be fulfilled in the precise design of these (non-small-molecule) therapeutics [63,64]. In general, the computer-aided projection of amino-acid-based therapeutics or candidate peptides [57] covers:

(a) peptidomimetics design (de novo design, peptide-driven pharmacophoric method, geometry-similarity method, sequence-based method, fragment-based method, hybrid peptide-driven shape, and pharmacophoric method);
(b) peptide design (ligand-based design, target-based design, and de novo design);
(c) the designing of therapeutic proteins (template-based design and de novo design).

CADD methods can be used in the initial structure proposal of these novel biopharmaceuticals, and the prediction of their properties (conformational features, stability, binding affinity, or interaction energies, for example), to study the mechanism(s) of interaction between them and the relevant biological targets (receptors), to predict the binding energies of the bonds formed during ligand–protein interactions, in order to explore the inhibitory activity of various physiological enzymes toward peptides and proteins [63]. In silico tools are employed in the design and evaluation of peptides showing anticancer, antihypertensive, antimycobacterial, anti-inflammatory, quorum-sensing, or cell-penetrating properties, as well as in the design and evaluation of peptide inhibitors for human immunodeficiency virus (HIV) or Alzheimer’s disease [58,64].

The heterobifunctional PROTAC molecule [46,59,65] consists of three parts:

(a) the target protein ligand (warhead)—the ligand (structural scaffold of a molecule of natural origin or a synthetic compound) targets proteins of interest (POIs); i.e., several nuclear receptors, various protein kinases, proteins involved in transcriptional regulation, neurodegenerative-related proteins, or fusion proteins;
(b) the E3 ubiquitin ligase ligand (E3-binder)—the ligand can be a structural scaffold of **lenalidomide**, **thalidomide**, or **pomalidomide** (so-called **LTP** agents), for example. These binders target E3 ubiquitin ligases, such as the Von Hippel–Lindau or cereblon (CRBN);
(c) a linker of varying size connecting the warhead and E3-binder—the linker contains a so-called anchor point influencing the length and steric properties (spatial arrangement) of a PROTAC therapeutic.

**Figure 2:**
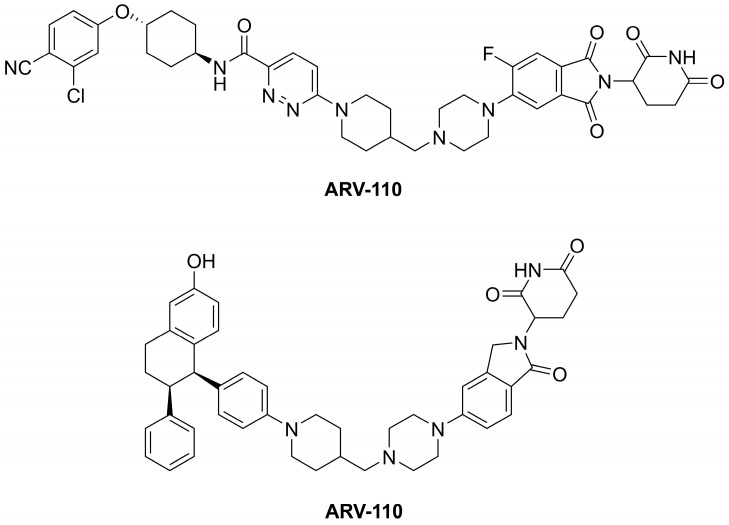
The structures of the oral-active PROTAC clinical candidates **ARV-110** and **ARV-471**.

PROTACs facilitate ternary complex formation between POI, which has to be selectively degraded, and an E3 ligase. The process results in polyubiquitination of the POI, and its subsequent degradation within a 26S proteasome. At least 20 PROTACs were involved in ongoing clinical trials by December 2022. Two of the most advanced oral-active PROTAC clinical candidates, **ARV-110** and **ARV-471** (Figure 2), are nuclear androgen receptor (**ARV-110**) and estrogen receptor-α (**ARV-471**) degraders for the treatment of prostate and breast cancer [66,67], respectively.

The online PROTAC-DB 2.0 database covers approximately 3300 PROTACs, 1000 ligands of POIs, 80 ligands of E3 ligases, and more than 1500 different linkers [68].

The designed and synthesized peptide-based PROTACs belonging to the first (older) generation contained a short peptide sequence. However, their activity was quite low, and they were characterized by poor cell permeability. Moreover, the size of such molecules allowed the immune system to recognize them and produce antibodies [69]. The new generation of PROTACs, i.e., small-molecule-based PROTACs, showed more promise, in terms of being designed and structurally optimized into the drugs. The scaffolds of small molecules incorporated into their structure as the moieties for recognizing an E3 ubiquitin ligase were more easily absorbed into the human body than the peptides were [69].

Generally, various types of degradation approaches can be considered, including PROTAC, in-cell click-formed proteolysis-targeting chimera (CLIPTAC), photochemically targeting chimera (PHOTAC), semiconducting polymer nano-PROTAC (SPNpro), floate-PROTAC, antibody-PROTAC conjugate, antibody-based PROTAC (AbTAC), ribonuclease-targeting chimera (RIBOTAC), transcription factor PROTAC (TF-PROTAC), chaperone-mediated protein degradation (CHAMP), biological PROTAC (bioPROTAC), or molecular glue [66].

The rational design and structural optimization of PROTACs with CADD techniques is extremely important, taking into strong consideration the main goal in the design of these innovative types of molecules—a precisely targeted protein degradation [46].

The virtual screening process requires several phases, including the proper selection of a warhead, E3-ligase, and E3-binder. The combinations of the warheads and E3-binders with specific linkers, available in existing libraries [68], or designed via generative algorithms, lead to a considerable number of structurally different compounds.

The discovery of suitable warheads and E3-ligands is similar to the process connected with small-molecule drugs. The design of linkers might be, in fact, complicated in the case of PROTACs because a particular POI and E3 ligase cannot interact with each other if an effective PROTAC molecule is not present. Thus, various properties of the linker, i.e., the length, the appropriate attachment site, the eventual incorporation of a photo-switchable group, or the selection of a clickable linker, have to be taken into very strong consideration [67].

Several docking procedures—more- or less-extensive MDy simulations—were employed to predict and design the ternary complex structures, and evaluate their stability [70].

PROTACs might be successfully utilized in the treatment of various diseases and disorders, including cancer [71], infections caused by bacteria [72] or viruses [73], neurodegenerative diseases [74], or inflammation and oxidative stress [75].

Gene therapy is a very powerful treatment modality for many diseases via the delivery of therapeutic nucleic acids to human cells [76]. This type of therapy involves the suppression of gene expression, artificially increased expression, or gene modification. The effective suppression of gene expression can be achieved at the mRNA level, i.e., post-transcriptional gene silencing, using RNA interference (RNAi) technology. The factor triggering this process is short dsRNA, in the form of microRNA (miRNA) or siRNA [76,77]. siRNAs, as the most promising type of RNA-based therapeutic oligonucleotide drugs, are double-stranded macromolecules, usually containing approximately 20–21 base pairs. These therapeutics, which confer a multitude of advantages compared to traditional treatment modalities, are used for gene downregulation or complete post-transcriptional silencing [61].

Proper chemical modification, improved efficacy, and targeted delivery using suitable carrier systems, which could minimize the off-target effects, immune response, and toxicity, are challenges within the development and optimization of siRNA-based therapeutics [78]. Indeed, in silico approaches could contribute to resolving those quests [79,80,81,82,83]. Various techniques of CADD were very helpful in attempting to find relevant therapeutic answers to coronavirus disease 2019 caused by severe acute respiratory syndrome coronavirus 2 (SARS-CoV-2; Betacoronavirus, *Coronaviridae*) [84,85,86,87], which was first identified in Wuhan (Hubei Province, China) in December 2019 [88]. Some examples of such in silico investigations are provided in the next section of the paper.

Both the *nucleocapsid phosphoprotein* gene and the *surface glycoprotein* gene in almost 140 SARS-CoV-2 strains spread worldwide were relevant biological targets for several siRNAs specifically designed in silico, using virtual modelling and docking analysis. The therapeutics targeted various conserved regions of those genes [89]. The research predicted that a group of investigated siRNAs might effectively fight a given RNA virus. Other siRNAs, proposed via molecular interaction and dynamics analysis, potently silenced an *RNA-dependent RNA polymerase* (*RdRp*) gene coding an essential viral RdRp enzyme [90]. The **siRNA-3** molecule, whose structure was proposed within a molecular docking analysis [91], could also be an effective anti-SARS-CoV-2 agent. In addition, the antiviral activity and safety of **siRNA-3** were confirmed in vitro, using a human embryonic kidney 293 cell line [91].

Thus, the idea to use siRNAs in the treatment of infections caused by various DNA viruses (such as the hepatitis B virus, herpes simplex virus type 1, human papillomaviruses, or murine herpesvirus 68), RNA viruses (such as coronaviruses, dengue virus, Ebola virus, hepatitis C virus, human respiratory syncytial virus, influenza virus A, rotaviruses, or West Nile virus) and retroviruses (such as HIV) is very reasonable [92,93,94].

Various siRNAs are currently approved worldwide for the treatment of diverse diseases, including neurodegenerative diseases [95], polyneuropathy [96], acute hepatic porphyria [97], hypercholesterolaemia [98], and primary hyperoxaluria type 1 [99]. Other siRNAs are also involved in clinical trials for the treatment of cancer [100] and immune-mediated diseases [101].

Traditional concepts in the design and development of drugs or drug candidates previously focused on their potency, without paying as much attention to their other characteristics [102]. Currently, the ability to predict the pharmacokinetic properties of drugs using appropriate computational techniques is a pivotal task in assisting early ADMET investigations. Properly chosen artificial procedures might considerably reduce the number of in vitro and/or in vivo tests in laboratories.

Solubility in an aqueous environment, permeability via the membranes of a biological system, metabolic stability, and transporter properties are of critical importance to the success of drugs in real life. These features notably influence their ADMET profiles [102].

The drug-likeness term is defined according to sufficiently acceptable features in clinically available drugs [103,104,105]. These characteristics include:

(a) structural and physicochemical properties—*MW*, lipophilicity defined via a logarithm of a partition coefficient value estimated/calculated for an octan-1-ol/water partition system (log *P*), effective lipophilicity, acid–base properties (acid–base dissociation constant; p*K*_a_), size, flexibility (number of aromatic and non-aromatic cyclic systems, number of double and triple bonds, number of rotatable bonds (*n*_rotb_)), number of carbon atoms, fraction of *sp*^3^ carbon atoms (number of *sp*^3^ hybridized carbons/total carbon count; *Fsp^3^*), distribution of electrons, polar surface area value (*PSA*; expressed in Å^2^ units), number of hydrogen bond donors (*n*_OHNH_) and acceptors (*n*_ON_), shape and stereochemical characteristics, reactivity, number of so-called heavy atoms, presence and number/absence of stereogenic centers, solubility, permeability, or chemical stability;
(b) biochemical properties—biotransformation, affinity to proteins, tissue binding, transport properties (connected with *PSA*);
(c) pharmacodynamics—the proper characteristics of the pharmacophore; the pharmacodynamic profile of a drug is notably influenced by structural and physicochemical properties, as well as its ADMET;
(d) pharmacokinetics and toxicity (ADMET) features—biological availability, drug–drug interactions, half-life, lethal dose values, proper characteristics of the toxicophore (the qualitative structural feature of a drug that is assumed to be primarily responsible for its toxic properties).

Many of the given parameters can be generated in silico using CADD procedures, various web engines, interactive applets, software, and software packages. For example, the log *p* value predicted via a computational CLOGP method [106] for an octan-1-ol/water partition system (CLOGP parameter), *n*_OHNH_, *n*_ON_, *n*_rotb_, as well as the *PSA* value [106,107] provide extremely valuable insight into the properties of drugs or promising drug candidates, including their ADMET features, and their ability to be passively absorbed when they are administered *per os*. In addition, this early information can help scientists and scientific teams to properly design their studies, make correct experimental decisions, and avoid wasting precious resources [54,108,109] within the synthetic phases of the research, via in vitro and/or in vivo biological assays. Detailed exploration of structure–property relationships is crucial to properly guiding structural modifications in order to make improvements in the considered features of the drugs or drug candidates.

The relevant descriptors defining the key structural, physicochemical, ADMET, and related parameters for individual drugs or various sets of (biologically active) molecules can be calculated through employing commercially available software or free web tools with an attractive design and user-friendly interface. A very good example of one such freely available online platform is the SwissADME predictor [110]. The limitation of SwissADME is the fact that only small-molecule drugs can be analyzed within its interactive environment.

Lipinski et al. (1997) stated [111] that the molecules characterized by *MW* > 500 Da, CLOGP > 5.00, *n*_ON_ > 10, and *n*_OHNH_ > 5 would be passively absorbed *per os* to a (notably) lesser extent. The small-molecule drugs, which were not substrates for active transporters and met the given criteria—rule of five (Ro5), i.e., *MW* ≤ 500 Da, CLOGP ≤ 5.00, *n*_ON_ ≤ 10, and *n*_OHNH_ ≤ 5—might be satisfactorily absorbed. Exceptions from Ro5 can be found mainly (but not only) among biologically active agents of natural origin [111,112]. More recent research [113] indicated that the *MW*, *n*_ON_, and *n*_OHNH_ parameters fitted well with how ligand-binding sites were formed in proteins.

Other in silico evaluations considered the appropriate balance between the structural rigidity and the flexibility of drugs whose characteristics were beyond Ro5 (bRo5), a favorable factor in their binding to biological targets with a high affinity. A balance in polarity was maintained if the calculated two-dimensional (2D) *PSA* was ≤250 Å^2^. The optimal lipophilicity of these bRo5 compounds was defined by a CLOGP of around 4.00. However, if their *n*_OHNH_ and/or *n*_ON_ were ≥6, the probability of sufficient passive oral absorption was reduced [108].

The oral delivery of peptide- and protein-based drugs is quite limited [114,115], due to their enzymatic digestion in the gastrointestinal tract, and insufficient permeation via the intestinal epithelium. The strategies for the oral delivery of those drugs [63,114,116], consider:

(a) their chemical modifications—particular changes are made according to empirical processes (PEGylation, glycosylation, the use of adjuvant molecules to enhance delivery and lipidization), as well as outputs from CADD (the optimization of aqueous solubility, for example);
(b) their modifications in formulation design—various permeation or absorption enhancers are used, for example;
(c) the modulation of a pH value of the environment;
(d) the direct inhibition of the enzymes responsible for therapeutics’ degradation (cleavage of peptide bond(s)).

Cyclic peptides with a limited conformational flexibility showed a better biological availability when being administered *per os*, compared to the linear ones. However, the criteria applied for the evaluation of the oral bioavailability of small-molecule drugs cannot be efficiently used within peptide drug development [117,118], in fact.

From the descriptors employed for the characterization of peptide therapeutics approved by U.S. Food and Drug Administration in the period 2012–2016, i.e., *MW*, CLOGP, *n*_OHNH_, *n*_ON_, *n*_rotb_, and *Fsp*^3^, only the last of these parameters met the relevant criteria for small-molecule drugs [118]. The most orally available peptides were described with *MW* ≤ 1200 Da and CLOGP = 5–8. In addition, they contained five times more *H*-bond donors and acceptors than was acceptable, according to Ro5, for small-molecule drugs [118].

Looking at the chemical structure of PROTACs indicates that they do not meet Ro5. When considering the *MW*, CLOGP, *n*_ON_, *n*_OHNH_, *PSA*, and *n*_rotb_ parameters, as well as the number of incorporated aromatic systems, the CRBN group E3 ligands (**LTP** ligands) predominate over the others. The reason for such a prioritization is a lower *MW* and more drug-like characteristics of these **LTP** agents. In fact, finding a way to achieve optimal pharmacokinetic properties is one of the major tasks in the design and development of PROTACs [65,119]. The newly introduced *AB-MPS* metric parameter was calculated for these therapeutics [65,119,120], using the calculated distribution coefficient (CLOGD) value, the number of aromatic rings, and *n*_rotb_. A lower *AB-MPS* score (*AB-MPS* ≤ 14) in a PROTAC indicated a higher probability that it would be orally absorbed.

Computational chemistry methods are also involved in various fields in medicine. Nanomedicine includes the processes of the diagnosis and treatment of a disease or traumatic injury, and its prevention in order to improve human health, using sophisticated medical therapies. These goals are achieved via molecular tools, and molecular knowledge of the human body [121]. Computational simulations valuably contribute to the design of medicinally utilized nanoparticles, the size of which varies from 1 nm to 100 nm, with optimized biological, functional, and toxicological characteristics. In silico approaches generate relevant computational models, which might help to design biomedical nanocarriers (liposomes, detrimers, gold nanoparticles, micelles, or virus-based nanoparticles, for example), and predict their interactions with the drugs loaded in them. Attractive smart drug delivery systems include nucleic acids, DNA, exosomes, or aptamers [122,123,124].

Artificial-intelligence-assisted nanomedicine rational design methods can be used in the detailed investigation or prediction of nanomaterials’ ability to cross biological barriers, and their biological properties. In addition, such design methods might reveal, and help us to understand more clearly, the complex interactions of the nanomaterials with biological systems and environments. A knowledge of the ADMET features of original nanoformulated drugs or drug candidates with high potency, selectivity, and specificity, using computational chemistry techniques, is also a crucial condition [122,123,125,126]. However, the importance of validation from obtained computational outputs with experimental data should also be mentioned.

There is no doubt that the various in silico approaches listed in this chapter will be increasingly employed in the prediction of human pharmacokinetics in early drug discovery. The accurate computational prediction of pharmacodynamics, ADMET, and pharmaceutical properties for various sets of compounds is currently possible. Such outputs from artificial tools are probably, at least, no worse than those related to the studies in vitro, with several decisive advantages—relatively rapid and dynamic evaluation processes, or the requirement of notably fewer resources [127], for example.

The most suitable approach to modern drug design and development is to create a properly balanced set of relevant in silico, in vitro, and in vivo descriptors for the simulation of the pharmacodynamic properties of the drugs and pharmacokinetics in specific human populations, to predict most accurately the interindividual variability [127,128].

## 4. Several Innovations within In Vitro Screening Approaches for Drug Candidates or Drugs

The traditionally established approaches for the proper testing of drug candidates or drugs could be very roughly divided into in vitro 2D culture screenings, in vivo evaluations, and in human studies [129]. Bioassay techniques can be classified [130] into two very robust subcategories, i.e., bench-top and primary bioassay screening, and high-throughput screening. The researchers try to continuously implement prospective innovative experimental steps and improved methodologies at each phase of the testing. Thus, several examples of such innovative in vitro approaches can be found in the following sections of the paper.

The conventional procedures for the lead optimization of anti-tuberculosis (anti-TB) drugs within the pre-clinical phase of their development are driven by the so-called three Ms; i.e., the determination of a minimum inhibitory concentration value; the efficacy determination, using murine models; and evaluation of the anti-TB activity in humans [131]. The way to optimize the in vitro screening of promising anti-TB compounds and, thus, the selection of anti-TB chemotherapeutic modalities, is to use more rational and dynamic non-standard assays, to more clearly understand where specific populations of mycobacteria reside. These compartments will be target sites for precisely selected and screened molecules. When combining in vitro conditions into one model, the researchers have to take into strong consideration the broth-based in vitro composition, changes in the pH value and oxygen levels during particular phases of the infection, or the availability of oxygen [132,133]. Hollow fiber systems allow for in vitro investigations into the pharmacodynamic/pharmacokinetic profile of a drug, or combination(s), and these evaluations are more consistent with in vivo pharmacokinetic studies [133].

The use of proper combination therapy, according to the data obtained from in vitro and in vivo experiments with optimally established conditions, can lead to the treatment of the disease in multiple compartments in the body. The result might be savings in the time and resources spent, shortening of the TB treatment, and minimizing the number of anti-TB compounds required to treat patients suffering from TB [134].

The innovative procedures within anticancer drug discovery and development are based on use of three-dimensional (3D) tumor models. These 3D methods, which more precisely mimic physiological conditions, as well as particular states of the disease, compared to their 2D counterparts, together with more advanced screening techniques and protocols, might lead to considerable improvements in the usefulness of in vitro investigations. In addition, they make the pathway from promising anticancer drug molecule candidates to effective, selective, and safe drugs for ‘real life’ more dynamic and more straightforward [135,136,137]. The classification system of 3D cancer models reflects different criteria and conditions. Thus, these models can be divided as follows: scaffold-free systems, organoids, and tumoroids as new avatars for drug screening, scaffold-based tumor models, hydrogel-based 3D cancer models, bioreactors, microcarrier-based models, and cancer-on-chip, respectively. More details regarding the specific characteristics of these 3D models can be found in a paper by Brancato et al. [135] or Booij et al. [136].

Human prion diseases are a class of neurodegenerative disorders that are regarded as incurable. The diseases are caused by a misfolded prion protein [138,139]. One of the main obstacles to finding relatively satisfactory therapeutic possibilities for humans has been the lack of well-defined and established screening methodologies. In future, the innovative and effective options within in vitro assays [140] would be the use of a protein misfolding cyclic amplification procedure, to successfully mimic the pathological process of conversion (misfolding) of a specific physiological prion protein (PrP^C^) into the abnormally folded one (PrP^Sc^). Another possibility is to carry out a very efficient real-time quaking-induced conversion amplification method, conceptually similar to the previous one.

## 5. Pre-Clinical In Vitro and In Vivo Studies

Earlier drug discovery was almost exclusively based on animal experiments, clinical observations, and serendipity. Nowadays, in accordance with the rational order of research, before it is approved for human use, a new compound must undergo a series of precise toxicological and pharmacological tests, which constitute a panel called “pre-clinical trials”. The first step is usually in vitro testing on genetically modified cell cultures, to check the drug’s effects on reproduction or carcinogenicity. In recent years, a major input has come from biochemistry. The effect of many drugs in human therapy could be explained biochemically as effects on specific enzymes or receptors. With the detection of more and more receptor subtypes, the activity spectrum of a single compound became more and more complicated. At present, molecular biology provides pharmacologists with human receptors and ion channels expressed in mammalian cells in culture. This avoids the apparent species differences, but the multitude of natural and perhaps artificial subtypes raises the question of physiological and pathological relevance. In recent years, a major input has come from biochemistry and molecular biology. The effect of many drugs in human therapy could be explained biochemically as effects on specific enzymes or receptors. The use of these methods allows for a much faster and more accurate characterization of the drug effect than that via traditional methods. At the same time, substances that cause side effects, or are not active enough, are omitted, resulting in a significant reduction in time and money. Predicting the pharmacokinetics of drugs or their side effects on the basis of a chemical structure is only approximately possible, which is why studies on animals or isolated organs are still a necessity. Similarly, effects seen in tissue cultures are often not typical of the whole intact organism. So, it will always be a challenge for the pharmacologist to correlate in vitro data with in vivo findings; according to the old saying: in vitro *simplicitas*; in vivo *veritas*. The main purpose of preclinical studies is to assess the safety of new drugs, and to confirm the direction of their therapeutic action. In vitro research—computer modeling, or tests on single cells or on cell and tissue cultures—has become more and more popular. Indeed, these studies are very useful in the initial phase, as they provide valuable information about intracellular processes, allow for initial toxicity testing, and help us to understand the mechanisms of action of new drugs. However, they do not cover the complexity of the whole organism, because individual cells react differently to the whole body. Furthermore, the possible side effects of drugs can be better detected in whole animals than in individual cells. Therefore, most of the results obtained in vitro must be replicated in vivo, i.e., in a living organism, which means that animal experiments are necessary for the discovery and evaluation of drugs [141].

However, they should be performed only if they are necessary and well-conceived. The credibility of the results obtained in pre-clinical in vivo studies depends mainly on the appropriate organization of the research, the selection of animal species, and their numbers and welfare. Well-designed studies makes it possible to extrapolate the results to humans. It is appropriate to conduct research on two different species, one of which is not a rodent. Therefore, in our laboratories, research is usually carried out on zebrafish (*Danio rerio*) and small rodents (mice and rats), also due to their low costs. However, a sufficiently large number of animals is needed in order to obtain a statistical result. In extremely important research, despite the high financial outlay, and other difficulties associated with it, substances are tested on dogs and primates. Another important issue is the selection of appropriate pharmacological tests and models. These tests and models must be relevant; that is, they should predict the intended therapeutic indications. A pharmacological model can be considered relevant or correlational if the effects obtained correlate with the results observed in human therapy. To be significant or “correlated”, a model must meet three basic criteria: firstly, the model must be sensitive in a dose-dependent fashion to standard compounds that are known to offer the desired therapeutic property; secondly, the relative potency of the known active agents in the model should be comparable to their relative potency in clinical use; and, thirdly, the model should be selective; that is, the effects of the known agents in this therapeutic indication should be distinguishable from the effects of drugs for other indications. Positive data with a new compound allow the prediction of a therapeutic effect in patients.

### 5.1. Rodent Experiments, the 3R Rule

Concerning pre-clinical studies, scientists can employ many models and tests, using rodents, to evaluate, among others, anxiety, depression, schizophrenia-like behavior, antinociception, locomotor activity and motor coordination, stress-induced abnormalities, epilepsy, or rewarding properties of newly-synthesized or natural products, e.g., drug candidates. After a pharmacological and toxicological screening, a non-toxic dose can be chosen for further testing, after many different routes of administration, e.g., intraperitoneally (i.p.) or subcutaneously (s.c.). The use of animals for experiments relating to the development of medical science has been an integral aspect for many years. This fact, however, often raises ethical questions, as, for the most part, interference with a living organism involves a violation of the welfare, i.e., the quality of life, of the animal. An experiment that is not carefully thought out can cause additional suffering, as well as pain, which is a strong stress factor [142]. Here, there is a conflict between the duty to look after people’s health, improve their social wellbeing, and benefit from science, and the environmental protection associated with preventing animal suffering [143]. In a report on the number of animals used for scientific purposes in the countries of the European Union, compiled in 1991 by the European Commission, we can find that this was 11.79 million animals in the 10 member countries. A 2008 report stated that this was now 12 million in the 27 member countries, which was followed by 9.58 million in 2017 [144]. The above data confirm the quantitative and qualitative development of alternative methods to animal models that has taken place in recent years. In some cases, these may even represent a better solution than studies conducted on mice and rats, the main model animals, whose body response often differs from that of humans. However, this is not always possible, and many studies still require the use of laboratory animals [145].

To reduce the number of unreflective in vivo experiments, and to make them more humane, in 1959, Rex L. Burch and William Russel developed the 3R principle, entitled ‘The principles of humane experimental technique’. It contains three guidelines:

- *replacement*,
- *reduction*,
- *refinement*.

These three rules involve increasing the use of alternative research methods to those using animals, reducing the number of animals used for scientific purposes altogether, and minimizing suffering, pain, and injury. At its core is an overall improvement in animal welfare, which can be understood as animals remaining healthy, well-nourished, and living in a friendly environment, possibly free from factors that are a source of stress or fear, and motivated to perform [146]. Considering the 3R rule, researchers take a more responsible approach to planning and conducting experiments [142]. Today, this principle is widely used by most countries in the world, and supported by organizations such as the World Organization for Animal Health, and the European Council [147]. The 3R principle also formed the basis for the European Parliament’s adoption of Directive 2010/63 regarding the protection of the interests of experimental animals. It now represents the standard for information on the use of animals for scientific purposes, as, in addition to members of the European Union, other independent countries have also brought this document into force, so we can say that the directive is implemented in two-thirds of European countries [144].

The first of the three Rs represents replacement. The aim of this is to use methods that do not require the use of animals, or to replace vertebrates with other research material, such as higher plants, vertebrate embryos, micro-organisms, or cell or tissue cultures. Examples include the use of human- or animal-derived tissues or cells, and the utilization of invertebrates, such as the fruit fly *Drosophila melanogaster*, or the worm *Caenorhabditis elegans*. Computer methods and mathematical models are also an alternative [142]. Nowadays, the possibilities for replacing animal models are increasingly promising. However, the in vitro system is much less complex than the organism, and does not offer the possibility of directly replacing the animal model, so combinations of different methods are often needed. These, however, do not always cover all aspects of the human biological system, with the result that tests conducted in vitro must often be repeated in vivo. Additionally, for the registration of medicinal products, prior animal experiments are required. These make it possible to determine the toxicity, teratogenicity, likely effects on the embryo, fetus, and offspring in subsequent generations. Therefore, although there is a legitimate need to replace laboratory animals in research, a universal solution has still not been developed that offers this possibility for every type of experiment [148,149].

Another rule of thumb is to reduce the number of animals used in a study. The use of too few animals, however, may lead to difficulties in the proper processing of the results, and the correct statistical analysis of the data obtained. Moreover, it is recognized that the suffering of each animal used should be minimized, even if this involves using more animals in the test. The answer to these concerns is to strike a balance between reducing the total number of animals allocated to an experiment, and obtaining reliable results. To achieve this, it is necessary to improve the design of the experiment itself, as well as the statistical analysis, by sharing results and materials between groups of investigators. To facilitate this process, various online tools are being developed, such as the Experimental Design Assistant website and application, which further assist researchers in designing and reporting their experiments. A reduction in the number of animals used by up to several tens of per cent can also be achieved through the use of new research technologies. They provide a source of objective and quantifiable data, as well as support with research [142,150].

A continuous improvement of research methods is at the heart of maintaining adequate welfare for laboratory animals. This includes the use of in vivo technologies that reduce pain and stress in animals. Examples include the ability to collect and analyze smaller volumes of samples, such as blood, plasma, or serum. This is particularly applicable in the case of small rodents, as it allows several samples to be taken from the same animal, thus minimizing the number of individuals in the group, and avoiding the need to kill the animal. Additionally, the use of analgesics, and habituating the animals to certain practices, can improve their welfare. However, improvement concerns not only the conditions under which the experiment is conducted, but also the conditions of maintenance. Therefore, the proper handling of the animals in husbandry and minor procedures is very important, due to the possible occurrence of stress in the animals, which, in further stages, may translate into differentiated experimental results [142,150]. The animals’ habitat itself, and the satisfaction of natural needs, are no less important as factors. For this reason, in the case of rodents, nesting material, toys, tunnels, or wooden elements are placed in cages as their habitat. Due to their herd instinct, if the specifications of the experiment do not indicate otherwise, the animals are reared in groups [151].

### 5.2. Zebrafish Model

As stated, various animal species are used in pre-clinical studies. Although mice and rats are the most popular, zebrafish are growing in importance.

*Danio rerio* (the zebrafish) is a freshwater tropical fish of small size (4–5 cm). Its natural habitat is rivers in India. The body is spindle-shaped and laterally flattened. The name (zebrafish) is associated with the presence of horizontal blue stripes on the sides of the body, resembling zebra stripes. There is sexual dimorphism. The male is slimmer, has golden stripes between the blue ones, and an orange color on the fins. The female has a larger, whitish belly, and silver stripes instead of gold. In addition, in adult females, a genital wart is located in front of the caudal fin [152,153,154].

Recently, there has been a tremendous increase in the use of the zebrafish in modelling human diseases, because of the numerous advantages it offers. Changes in the law to reduce the suffering of animals during scientific research have made the zebrafish a popular alternative to previously used mammals [150]. This is due to the ease of breeding zebrafish, their rapid development, and their ex vivo fertilization.

For scientific purposes, zebrafish are grown in laboratory aquaculture under controlled conditions. These animals, before being sent for scientific research, obtain a health certificate confirming the fact that they are free from disease. Despite this, all fish are quarantined prior to testing [155]. Following ex vivo fertilization, there is the rapid development of a translucent embryo, with developed segmental muscles, organs, and a brain beginning to form. After 48–72 h, a free-floating larva hatches, transforming into a juvenile, which reaches sexual maturity after about 3–4 months. *Danio rerio* lay about 200 eggs a week. The eggs develop externally, and are small (ca. 4 mm at 5 days post-fertilization, dpf). This makes it easy to array embryos and larvae in 96-well microtiter plates for high-throughput drug screening [152,156]. The embryos are translucent, which allows the microscopic examination of very early developmental processes that cannot be observed in mammals, due to our development inside the uterus [157]. As vertebrates, these fish show a similarity to humans in their tissues and organs, and the processes taking place in them. Of particular importance is the analogy in the structure of the brain and the nervous system, and their functions. Recently, the zebrafish has emerged as a valuable and attractive model for various neurological disorders, including schizophrenia [158,159]. These fish have the ability to regenerate cells and tissues: brain, spinal cord, heart, retina, photoreceptors, and fins. Research is being undertaken on the possibility of using this ability in retinal diseases of various etiologies [160]. Another important feature is the convergence of the zebrafish genome with the human one. *Danio rerio* has 25 chromosomes with an approximately 70% homology to human genes. The genetic information of zebrafish can be modified. The resulting phenotypically mutated individuals correspond to true human disease states, and include ethology, disease progression, and resolution. This has contributed to the wider use of these fish in pre-clinical studies of various diseases with the use of new chemicals and existing drugs in indications other than the original ones. These studies can be performed on both embryos and larvae. This gives the opportunity to better understand the action of the drug, determine its pharmacokinetics and pharmacodynamics, and obtain the necessary information for the next stages of research [156]. Numerous advantages of *Danio rerio* have allowed the creation of models of many diseases. Several cancer models have been created, including leukemia, melanoma, cardiovascular disease, the immune system, infectious diseases, diabetes, obesity, and behavioral disorders [161,162]. This has contributed to progress in many fields of science, including oncology and toxicology. New studies with zebrafish and new substances with medicinal properties are being modelled all the time [161]. The disadvantages of these models include problems with the standardization of the test and its reproducibility, as well as problems with the administration of the substance. Most often, they are dissolved in the water in which the fish swim. This poses a risk to them, and may also pose a problem with regard to the data obtained for larger vertebrates [163].

Numerous advantages of zebrafish have made them more and more often used in pre-clinical and toxicological studies on various substances. The versatility of this model has contributed to significant developments in biology, medicine, and pharmacology, as well as the discovery of new drugs, and new applications for those already known [164].

## 6. Clinical Trials—General Information

Promising results of pre-clinical studies allow the drug to be admitted to the next stage of testing—clinical trials, which are carried out on human volunteers. The purpose of clinical trials is to assess the pharmacological and/or pharmacodynamic effects, and to determine the adverse events (AEs), of the tested chemical compound. Clinical trials are conducted in accordance with the Good Clinical Practice (GCP) guidelines developed by the International Council for Harmonization (ICH). GCP is a set of legal and ethical standards that strictly defines the method of planning, conducting, monitoring, documenting, and reporting clinical trial results. In the clinical trial planning phase, an independent bioethical committee must issue an opinion on the project, and each subsequent stage of the trial must be carried out with the utmost care for the patient’s wellbeing. The superior document in clinical trials is the clinical study protocol, which defines the organization and course of the trial, as well as all procedures that should be performed as part of it. A patient (referred to as a study participant in a clinical trial) voluntarily joining a clinical trial is required to sign the Informed Consent Form (ICF), the second most important trial document, which defines, among other things, the trial participant’s rights and obligations, as well as the risks and benefits associated with the trial. It should be emphasized that the study participant enters the clinical trial fully consciously, and the new drug (referred to as the investigational product or study drug) may, but does not have to, help to alleviate the symptoms of the disease. Therefore, the study participant has the option to withdraw from the study at any time, without giving a reason [165].

**Figure 3:**
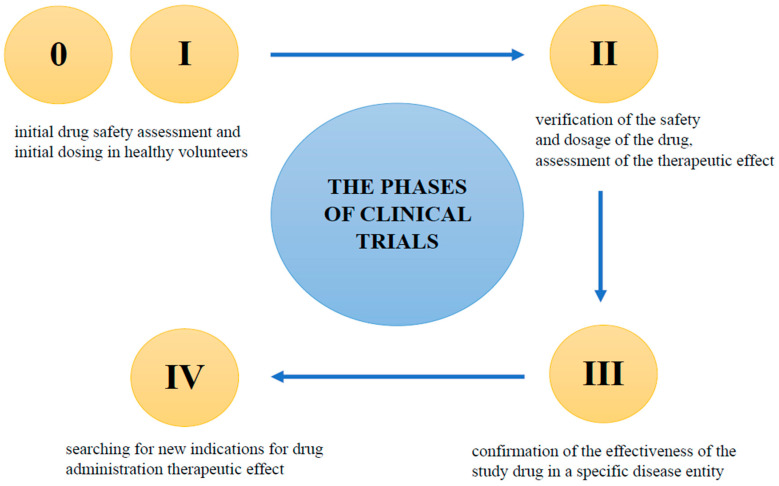
The phases of clinical trials.

Most clinical trials are randomized, which means that study participants are randomly assigned to treatment groups: each person has the same probability of being assigned to a study drug or placebo cohort. In open-label clinical trials, both the patient and the physician (referred to as the “Investigator” in clinical trials) know what substance is administered, but this may affect the reliability of the results. Therefore, most studies are double-blind, which, in practice means that neither the study participant nor the investigator has any information as to which treatment group the participant has been assigned to [165]. There are five main phases in clinical trials (Figure 3).

**Phase 0**

These are preliminary human studies to confirm the results of pre-clinical studies. Subtherapeutic doses of the drug are used on a small group of healthy volunteers. Phase 0 studies are aimed at deepening information on the mechanism of action of the drug, and the processes it undergoes after entering the body. This phase of clinical trials typically involves a group of between 10 and 15 healthy volunteers. However, it is often skipped in favor of Phase I clinical trials [166,167].

**Phase I**

If Phase 0 is skipped, this is the first-in-human (FIH) phase. It is carried out on healthy volunteers, usually men. In the event that the study drug may be potentially toxic (e.g., in cancer), the phase I study is carried out on sick people. This part of the study is primarily used to assess the safety of the drug, and determine the expected dosage. Phase I studies also provide valuable pharmacokinetic data on absorption, metabolism, excretion, and potential interactions. Typically, between 50 and 100 healthy volunteers participate in this phase of clinical trials [166,167].

**Phase II**

The next stage of clinical trials concerns people diagnosed with the disease. Patients must meet all of the inclusion criteria in the study protocol, and not meet any of the exclusion criteria. The protocol assumes, among other things, what age potential patients should be, what diseases they can/cannot suffer from, and what concomitant medications they can/cannot take. Phase II usually involve a small number of patients, and the main purposes of the trials are to confirm the safety of the investigational product, and to obtain data on its effectiveness. This phase usually involves a group of between 100 and 300 patients who are suffering from the disease [166,167].

**Phase III**

The success of Phase II enables the next stage of testing, which is Phase III. Studies are carried out on a larger population, and in many clinical research centers around the world. The duration of Phase III varies, and depends on the information contained in the protocol; usually, the study lasts several years. The purpose of this research is to determine the optimal dose, and to confirm the effectiveness of the study drug in a much larger group of people, from several hundred to several thousand patients. A positive result in Phase III enables the registration of a new drug [166,167].

**Phase IV**

Phase IV is the time after the drug is placed on the market. This phase of studies allows for the assessment of efficacy and AEs regarding the long-term use of the drug by thousands of patients around the world. During the observation of drug activity, the results of the previous phases of the study are verified. New indications for drug administration are also sought [166,167].

## 7. Conclusions

Drug research on new, potentially effective drugs, including vaccines, is one of the goals of the medical sciences, and constitutes an integral part of national and transnational health support programs. Analyses across all therapeutic areas indicate that the development of new medicine, from molecule synthesis, to target identification and approval for marketing, takes over 12 years [168]. Despite advances in biotechnology, this process is lengthy, costly, and difficult. Any advances or progress in science and biotechnology immediately find an application in medicine, in pharmacy, and in drug discovery and development. Investments in drug design are worthwhile, because the better a given drug candidate is designed during the experimental stage, the less likely it is for the drug to fail in the late stages, where the tests are more expensive, especially in clinical trials, and the safer and more effective it will be. The COVID-19 pandemic forced scientists to rethink how to accelerate the timelines of the discovery and development of drugs and vaccines, as an example. Modern, effective, and less costly methods for drug discovery are required, including artificial intelligence, which is able to gather and analyze large amounts of data in a short time, to select appropriate targets and complementary ligands, to design and perform tests. The ultimate goal for future drug development is to be able to design and develop a specific, non-toxic, effective, and patient-tailored drug over a period of several hours. Although this goal seems fantastical at the moment, it is completely achievable in the near future.

## Data Availability

Data sharing is not applicable.
